# Letter from Dr. Bosworth, of Paris

**Published:** 1869-12

**Authors:** A. W. Bosworth

**Affiliations:** Paris


PARIS, *Nov.* 19, 1869.

MR. EDITOR: It may be interesting to some of your readers to know what takes part of the attention of the medical profession in Paris.

At the Academy of Sciences, presided by M. Claude Bernard, on the 2nd of November, M. Bouchut gave the following statements concerning the hydrate of chloral — formed by union of chlorine with alcohol. He said that it is a powerful sedative of the motor and sensitive nervous system. If it is not crystallized and very pure, so as to disengage, under the influence of Potassium, vapors of chloroform without coloring the liquid, it is unfaithful, and may be very dangerous.

The dose ought not to be more than 5 grammes for an adult, for children, must begin with 1 or 2 grammes. (The French gramme is about 20 grains English.) It can be administered by the mouth, or by the rectum, which produces the same effect as when introduced into the stomach. It is dangerous to use it for hypodermic injection. Arterial tension augments under the influence of sleep produced by this medicament; the pulse, at the same time, becomes more frequent, and diminishes after waking.

Its absorption by the rectum takes place sooner than by the stomach.

The urine produced by it during sleep is neutral, and boiled with Fehling's solution does not reduce the salts of copper; but, twenty-four hours after waking, when the urine then contains chloral, it becomes more dense and reduces the copper salts. At this moment we might be led to consider that there was a temporary glycosuria which does not exist.

The action of chloral is similar to that of chloroform, but is slower to be produced, and lasts much longer. It produces, with certain patients, a muscular and moral agitation resembling alcoholic intoxication, but has nothing disagreeable or disgusting.

With nearly all it produces sleep, rarely accompanied with hyperesthesia, and, in the great majority of cases, is remarkable for a very strongly accentuated anæsthesia. This latter is in proportion to the dose; this being from 2 to 5 grammes, according to the age, produces complete anæsthesia, and permits, without pain, cauterization with Vienna caustic, extraction of teeth, etc.

As a therapeutical agent, it is a sedative of the violent pains of gout, of nephritic colic, of dental caries; in fact, is the first of anæsthesias administered by the stomach.

It is the most prompt and efficacious remedy to employ in intense chorea, when we wish to make rapidly cease that agitation which, of itself, menaces the life of the patient.

M. Personne, experimenting on dogs with this medicament, has found that the chloral partially transforms itself into chloroform, by the influence of the alkalinity of the blood; and that after the administration of pure chloral, its presence can be chemically recognized in the blood and in the other fluids of the economy.

M. Dumas confessed, at the same meeting, that he, as well as Liebig and other distinguished chemists, had long known this chloral, without having ascertained its physiological and therapeutical properties.

On the 20th of September, M. Demarquay said, at the Academy of Medicine, that in twenty cases experimented on, six were negative. In fourteen cases where sleep was complete (twelve women and two men), in was produced in fifteen to thirty minutes. He gave 1 gramme in a spoonful of the sirup of the balsam of tolu. There were no accidents. That sleep is slight, and resembles not at all that produced by chloroform. That the least noise wakes the patient, but directly he falls asleep again. That their sleep was troubled with dreams and hallucinations. That the smallest pricking, a simple pressure, causes the patients to complain, and they immediately withdraw the part of the body touched. That chloral is not in the least an anæsthetic.

The assertions of this gentleman, we must remember, were made two months ago, and that he only employed small doses.

M. Bouchut, who certainly is good authority, distinctly declares the medicament to be a very strong anæsthetic when employed in sufficient doses.

*Aqua Puncture.*—Such is the name of a new and ingenious method of revulsion, created by M. Mathieu, one of the most skillful manufacturers of surgical instruments in Paris. It consists of a forcing pump, having attached to it a metallic tube, terminating in its further extremity in a filiform manner, thus allowing, by pressure on the lever of the pump, a capillary jet of pure water to flow with sufficient force to penetrate the cellular tissue beneath the skin. This puncturing of the skin causes a slight, white elevation, from the centre of which sometimes flows a drop of blood. The first pain, caused by the puncturing of the skin, is quite severe, causing the patient to start up pretty briskly. He will sometimes allow fifteen or twenty punctures to be made at one sitting. In less than half an hour the water injected has been absorbed, and the pain has gradually disappeared.

I have seen this ludicrous method employed on a large number of patients at the genito-urinary clinic of Dr. Mallez. It certainly causes various muscular and neuralgic pains to disappear, at least for a certain time, better and quicker than many other revulsives. M. Mathieu relates some cases of permanent cure of these disagreeable pains. Although the instrument is just getting introduced into several of the private cliniques in Paris, and into no hospital as I have seen, yet the inventor says he used it some three or four years ago. It is a nice instrument to have in one's office, as it amuses the patients, does them no harm, and often relieves them very much. As this article is more therapeutical than otherwise, allow me to terminate by noticing another practical instrument just invented by M. Deulafoy, a young Paris doctor.

It was presented at the Academy of Medicine, November 2, and consists of a long canular-trocar, attached to a pump. The canula are minute and long, so as to be thrust through any tissue or organ in search of fluid, and at the same time allowing no entrance of air. The seat of an abscess, the presence of urinary, serous or purulent effusion, may thus easily be ascertained, and its nature determined by microscopical examination.

It is useful not only for diagnosis, but also for treatment, as injections are readily made with it.

As suction can be made, fluid can surely be withdrawn, when the ordinary capillary canula still leaves the surgeon in the dark as to diagnosis, for reasons too clear here to be mentioned.

We augur for this instrument a very favorable reception.

A. W. Bosworth, M.D.

